# The Book World of Medicine and Science

**Published:** 1897-08-21

**Authors:** 


### THE BOOK WORLD OF MEDICINE AND SCIENCE.

Sanitary and Social Questions of the Day. By An Observer. (London: Cotton Press, Granville House, Arundel Street, W.C. Demy 8vo. Cloth, 2s. Pp. 92.)

This book consists of a collection of articles contributed to medical periodicals. The range of subjects is somewhat wide. Part I. consists of essays turning more or less on matters educational, two of which, "The Mollusc and the Child" and "In Defence of the Birch," especially appeal to us. The second part deals with questions of sanitation. The gist of the whole series may be summed up into a plea for more room, more facility, and more liberty for the natural development of the human organism. A readable book is, of necessity, one which has something in it to awaken curiosity and thought, and this is a readable book on these grounds. There is a serene discursiveness yet absence of dulness about it which gives one a pleasant smack of Oliver Wendell Holmes, and it is probably in this quality, rather than in anything that the book has to say, that justification both for its existence and reappearance lies.

A Contribution to the History of Respiration in Man. By William H. Marcet, M.D., F.R.C.P., F.R.S. (Illustrated. Pp. 116. London: J. and A. Churchill. 1897.)

The subject of these lectures has been a matter of close investigation by the author for over twelve years. His experiments have been conducted not only in the laboratory but very largely in the High Alps, and many difficult and abstruse points have been elucidated by his painstaking and scientific labour. We have no hesitation in saying that such a collection of observations will prove of great value and interest to those who desire to be fully informed on the physiology of respiration, although appealing mainly to deep thinkers and to those who value scientific data based on elaborate and careful work.

The Swedish System of Physical Education. By Theodora Johnson, Principal of the Swedish Institute, Clifton, Bristol. (Bristol: John Wright and Co. 1897. Price 3s. 6d. net.)

This is not a handbook or an attempt to teach the system recommended. It is a book written in praise of the Swedish system, so-called, of physical education, with sufficient details interspersed to give the uninitiated some idea what it is like and what it aims at. A number of particulars are given about Ling, his life, and his theories, or, at any rate, the ideas which he endeavoured to put into practice. Then a description is given of the principal divisions into which the various movements used are classed, and of some of the forms of apparatus employed in the gymnasium. Then we come to medical movements and massage, where we find a sketchy account of some of the more ordinary manipulations employed in massage, and many statements as to the beneficial effects which may be expected from this process when skilfully applied. Great stress is laid on the latter condition, and upon the time and diligence which must be expended before anyone can become qualified to practise the art with full efficiency. Next come a series of short outlines of the sort of cases in which the system recommended may be used and the sort of cures which may be expected, sketches which might almost as fitly be described as testimonials to the efficacy of the treatment. Then come, as was inevitable, a chapter on spinal curvatures and more cases. It does not appear to us that this little book is likely to be of any particular service to medical men, but it is a clever book and will excite the interest of its readers in the system described, and this is no doubt the object the author had in writing it. What we feel in regard to these and other forms of movement cure and gymnastic exercises is that they are worthy of very careful study by medical men. There can be no doubt about the therapeutic uses of regulated muscular action in many morbid conditions, the difficulty is to get it properly applied and to get its management undertaken by proper people. We cannot for a moment admit that it takes five years' work to develop a proper massage hand, or that it is impossible for a medical man if he so chooses not only to supervise, but even to perform, many of the manipulations which he may find necessary. A practical knowledge of all means of cure certainly falls within the province of the physician.

The London Manual, 1897-98. Edited by Robert Donald. (London: Horace Marshall and Son. Price 1s. 6d.)

The justification for the publication of this manual is to be found in its first sentence, "There are over four hundred public authorities at work in governing London and they spend over twelve and a-half million pounds a year." So numerous, in fact, are the authorities engaged that there would seem to be justification for publishing an even more elaborate handbook than the one before us. What, no doubt, the publishers have seen is that a work of this sort, if it is to be of real utility, must be up to date, must be frequently republished, and therefore must be reasonably cheap. The amount of information squeezed into its pages is surprising. Maps are given showing various details; the different areas included in the municipal, poor-law, police, and postal London; distribution of political parties at the last election and at the County Council election; the areas supplied by the various water companies, &c. The subjects dealt with are far too numerous even to mention, but in all cases the information is systematically arranged and is given, as far as is possible, in an exact numerical form. At the end comes a complete directory of members of public bodies within the area of the county of London, giving the name and address and the titles of the public bodies with which each person is connected. Altogether, the work is likely to be of very great utility to those engaged in the government of London, and that this means no small number may be understood from the fact that the directory above alluded to covers 69 closely-printed pages.

